# The view of severely burned patients and healthcare professionals on the blind spots in the aftercare process: a qualitative study

**DOI:** 10.1186/s12913-015-0973-2

**Published:** 2015-08-01

**Authors:** Wendy Christiaens, Elke Van de Walle, Sophie Devresse, Dries Van Halewyck, Nadia Benahmed, Dominique Paulus, Koen Van den Heede

**Affiliations:** Belgian Healthcare Knowledge Centre (KCE), Kruidtuinlaan 55, 1000 Brussels, Belgium; Möbius, Kortrijksesteenweg 152, 9830 Sint-Martens-Latem, Belgium

**Keywords:** Burns, Burn units, Organisational policy, Aftercare

## Abstract

**Background:**

In most Western countries burn centres have been developed to provide acute and critical care for patients with severe burn injuries. Nowadays, those patients have a realistic chance of survival. However severe burn injuries do have a devastating effect on all aspects of a person’s life. Therefore a well-organized and specialized aftercare system is needed to enable burn patients to live with a major bodily change. The aim of this study is to identify the problems and unmet care needs of patients with severe burn injuries throughout the aftercare process, both from patient and health care professional perspectives in Belgium.

**Methods:**

By means of face-to-face interviews (*n* = 40) with individual patients, responsible physicians and patient organizations, current experiences with the aftercare process were explored. Additionally, allied healthcare professionals (*n* = 17) were interviewed in focus groups.

**Results:**

Belgian burn patients indicate they would benefit from a more integrated aftercare process. Quality of care is often not structurally embedded, but depends on the good intentions of local health professionals. Most burn centres do not have a written discharge protocol including an individual patient-centred care plan, accessible to all caregivers involved. Patients reported discontinuity of care: nurses working at general wards or rehabilitation units are not specifically trained for burn injuries, which sometimes leads to mistakes or contradictory information transmission. Also professionals providing home care are often not trained for the care of burn injuries. Some have to be instructed by the patient, others go to the burn centre to learn the right skills. Finally, patients themselves underestimate the chronic character of burn injuries, especially at the beginning of the care process.

**Conclusions:**

The variability in aftercare processes and structures, as well as the failure to implement locally developed best-practices on a wider scale emphasize the need for a comprehensive network, which can initiate transversal activities such as the development of discharge protocols, common guidelines, and quality criteria.

## Background

Burns range from minor burns to devastating injuries. The severity depends on factors such as age, depth and surface area of the lesion, body region, simultaneous smoke inhalation and previous health conditions [1]. Different classification systems exist: systems based on mechanism or cause, the degree or depth of the burn or the ‘Total Body Surface Area’ (TBSA) burned are the most common ones [2, 3]. Several national guidelines define “patients with severe burn injuries” [4–6] according to (overlapping but often slightly different) criteria for referral to a burn centre (e.g. burn injuries children >10 % TBSA). Since the nineteen sixties, burn centres focused mainly on the acute, critical care phase. This approach contributed substantially to the decreasing mortality rate among patients with burn injuries. At this moment even patients with severe burn injuries (>80%TBSA) have a realistic chance of survival in developed countries [7].

Burns cause long-term discomfort, functional impairments and psychological problems. A severe burn injury can impact on all aspects of a person’s life, including their aesthetic appearance, financial situation, relationships with others, psychological, social (e.g. integration work/school) and physical functioning [8]. As a consequence there is a need for long-term specialist physical, social and psychological rehabilitation to enable burn patients to live with this major bodily change [7, 9].

The rehabilitation process should begin at admission to the burn centre [10]. The care process after discharge from the acute burn centre is often referred as ‘aftercare’ [7]. This typically includes outpatient visits in hospitals (mainly in burn centres) and primary care services provided by e.g. nurses, physiotherapists, general practitioner, psychologists. However, the care for burn patients is not restricted to these burn centres or the aftercare services. In some cases, a transition phase is needed between the burn centre and out-patient care services. This can, for instance, include the treatment in step-down units (e.g. paediatric unit, general surgical unit) or the referral to specific centres for multidisciplinary rehabilitation.

In general we aimed at collecting information on the current problems and bottlenecks in the organization of aftercare for patients with severe burn injuries and explore solutions for a more effective system. From the point of view of the patient we wanted to know the specific problems encountered during aftercare, as well as their unmet care needs.

## Methods

Qualitative research methods are especially suited to identify problems and unmet needs, since they allow for an in-depth understanding [11]. In order to learn from both the patients' and health care providers' perspective, both patients and care providers have been interviewed. Individual face-to-face interviews were used to explore patients’ views on and experiences with the care process as well as to explore the views of physicians and representatives of patient organizations on the aftercare process. Individual interviews enabled respondents to talk freely as anonymity was guaranteed.

Allied healthcare professionals were interviewed in focus groups because we were interested in the interaction emerging from a group of care professionals with varying work experiences determined by the variety in professional background (physiology, nursing, psychology) and hospital setting. Also, since these care professionals are used to meet for other purposes (e.g. organization of burn camps; organization of training sessions), a culture of trust was already installed, and individual anonymity was not felt to be necessary to facilitate the openness of the participants. In each group multiple disciplines (nurses, physiotherapists, social workers and psychologists) were represented.

### Setting

In Belgium, there are six burn centres with a total of 70 beds to take care of patients meeting the legal criteria for admission or referral to a burn centre (see Table 1). Five out of six burn centres exist as wards within general hospitals (three of which are academic hospitals), the sixth burn centre is part of a Military hospital although most treated patients are civilians. All six centres typically focus on the acute care phase. There is, however, variability between centres regarding the part of the care process in which they intervene. Some centres care for the patients from admission until discharge or return to previous place of residence. Other centres care for the patients in the acute phase only and have patients transferred to medium care units (e.g. general surgical unit or paediatric unit) before final discharge [12].

**Table 1:** Legal criteria for admission or referral to a Belgian burn centre (if one of the 11 criteria apply, patients can be admitted in a burn centre)

1	Second degree burns larger than 10 % of the TBSA and third degree burns for patients up to 10 years old or more than 49 years old
2	Second and third degree burns larger than 20 % TBSA
3	Third degree burns larger than 5 % TBSA
4	Significant burns that involve face, hands, feet, genitalia, perineum or the major joints
5	Electrical or chemical burns
6	Inhalation injuries (Bronchi, Alveoli, …)
7	Burn injuries in patients with pre-existing medical disorders that could complicate treatment management or influence the recovery or the mortality
8	Burn injuries in patients who will require special social or emotional intervention, including neglected or abused children
9	Burn associated with concomitant significant trauma or burn with great local complications
10	Lyell syndrome (toxic epidermal necrolysis or Staphylococcal scalded skin syndrome)
11	Major traumatic or medical epidermal necrolysis (gangrene, necrotizing fasciitis,…) larger than 10 % TBSA

The burn centres are characterized by highly specialized multidisciplinary teams. They focus on the acute care phase but also have a role in the aftercare process. After a large industrial disaster (July 30, 2004, Ghislenghien: gas explosion resulting in 132 casualties, 24 deaths and 25 severely burned), the legislator created two new positions in each burn centre with the primary aim to improve aftercare services: care coordinator and psychologist (0.5 Full-time equivalents per centre each). The initiators envisaged that the function of care coordinator would play a pivotal role in multidisciplinary discharge planning, follow-up and in the prevention and monitoring of drop out. Nevertheless, the absence of specific requirements or a clear job description resulted in substantial variability in the way this function was implemented in terms of professional background (e.g. nurses, physiotherapists, psychologists) as well as in terms of role specification (e.g. focus on wound care versus focus on coordination of the entire multidisciplinary plan). In addition, some centres divide this 0.5 FTE position into smaller FTE portions attributed to several caregivers or increased the FTE on their own budget. Other centres have one care coordinator. Also the implementation of the 0.5 FTE psychologist per centre varies (e.g. focus on in- and/or outpatient care) between centres [12].

### Study sample

Patients, physicians, allied health professionals and representatives were selected based on a purposive sampling approach.

#### Patients

The minimal selection criteria for inclusion were:

- Being 6 to 24 months post burn injury. This period was chosen to balance experience with aftercare and recall bias;
- History of hospitalization in one of the six burn centres and meeting the legal criteria for admission to a burn centre. Patients with ‘Lyell syndrome’ (criterion 10) are out of scope of the current study since these patients have very different aftercare needs.

In addition to these minimal criteria, we sought variation in age, gender, having undergone surgery, visibility of the scars, financial or psychosocial precarious situations, and region. Characteristics of Patients are summarized in Table 2.

**Table 2:** Sample description

Patients (and parents)^a^	
Parents of children <12 years	8
Adolescents between 12 and 18 years	3
Parents of Adolescents between 12 and 18 years	3
Adults between 18 and 30 years	3
Adults between 31 and 40 years	1
Adults between 41 and 65 years	8
Adults older than 65 years	3
*Sub-Total*	*29*
Physicians	
Plastic surgeons	5
Anaesthesist	1
Rehabilitation medicine	1
*Sub-Total*	*7*
Burn care organisations^b^	
Patient organisations	*4*
Allied health professionals	
Care coordinators	4
Physiotherapists	3
Nurses	4
Social workers	2
Psychologists	4
*Sub-Total*	*17*
Total	57

^a^For each of the age groups additional criteria were pre-defined such as gender, number of surgical interventions, presence of visible burn injuries, psychosocial problems, financial problems. The detailed table can be found elsewhere

^b^Note that the interviews were in Dutch but that two of the largest charity organizations work on a national level (targeting patient residing in the Dutch- as well as in the French-speaking regions of Belgium)

Patients were recruited by the care coordinators who sent invitations and an informed consent form with a numerical code to a selection of patients who met the inclusion criteria. They were asked to contact the research team (by phone or pre-stamped envelope) if they agreed to participate. For each numerical code the care coordinators sent a profile description of the patient. Once the researchers received the informed consent form, the code could be matched with the profile description provided by the care coordinators. The research team contacted patients to organize face-to-face interviews. Doing so, researchers could keep track of which patient profiles were already included and which were still missing at each point during recruitment without knowing the identity. Additionally, care coordinators could not trace back which patients actually agreed to participate.

#### Physicians

An invitation for an interview was sent to the responsible physicians of each of the six burn centres as well as to the responsible physician from a rehabilitation centre that takes care for patients with severe burn injuries.

#### Representatives of organizations for patients with burn injuries

We identified four Belgian patient organizations specific for burn injury. From each organization one representative was invited.

#### Allied health professionals

A sampling grid was used in order to achieve a sample with a balanced number of participants for each burn centre representing the key professional groups/functions (i.e. physiotherapists; social workers; psychologists; nurses; care coordinators). Desk-research, key informants from our existing network and punctual information were used to compile this list. Out of this list, people were invited to one of the two focus groups, one in Dutch and one in French.

### Data collection

#### In-depth interviews

The interviews were conducted between January and April 2013. They lasted between 1.5 and 2 h and the location was chosen by the interviewee. All interviews were audio-recorded and transcribed verbatim. In the case of the patient interviews, the informed consent was explained and signed by interviewee and interviewer.

**Fig. 1:**
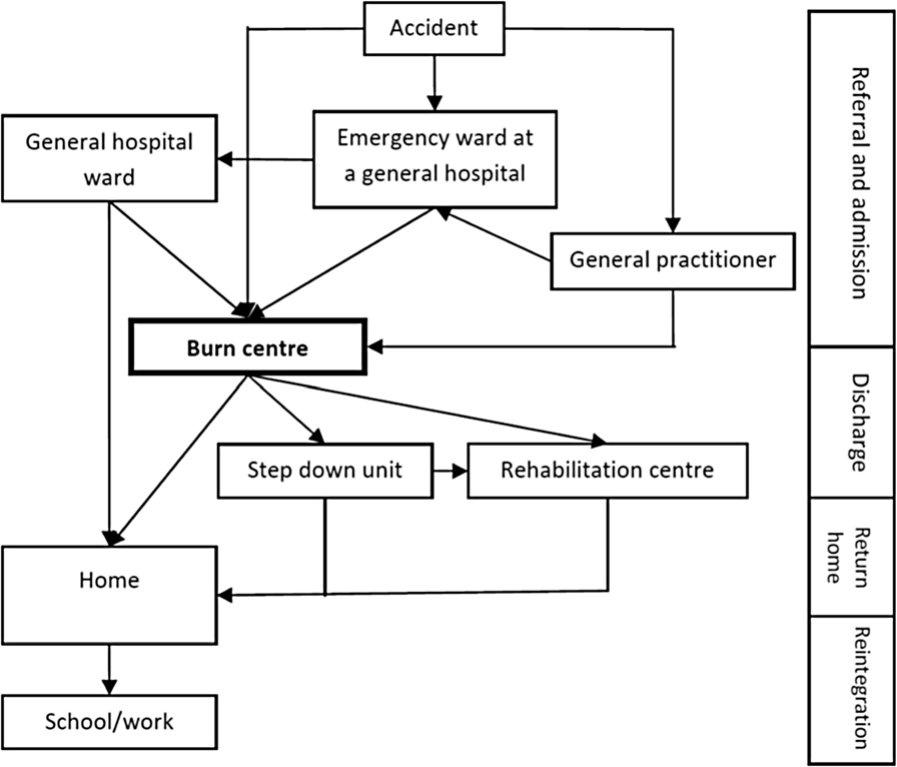
Severely burned patients’ care trajectories and transitions in the care process

Interview guides (i.e. for adult patients; adolescents; parents of adolescents; parents of young children; physicians and representatives of patient organizations) were developed for the in-depth interviews and are available upon request. The research team based the questions on information obtained during site visits^1^ of the six burn centres and a scoping review of the literature on unmet needs in the aftercare for patients with severe burn injuries [12]. The interview guide was built around the main transitions in the care process (see Fig. 1): referral and admission to the burn centre, discharge, return home, reintegration in social life.

The interview guides for patients were tested during 4 test interviews. (2 Dutch: 1 parent, 1 adult; 2 French: 1 parent and 1 adult). The test interviews were observed by two members of the research team behind mirror glass. The test interviews allowed the research team to adapt the interview guides. The data collected during the test interviews were not included in the analysis.

#### Focus groups

The two focus groups, each lasting 2.5 h, were held in February 2013 by 3 persons: a moderator who lead and synthesized discussions; an observer who took notes on the non-verbal communication in the group and helped the moderator to encourage participants to talk; a reporter who took notes on the discussion [13]. Before starting the focus group interview, the objectives, the speech distribution rules and the roles of the moderator, observer and reporter were explained, confidentiality of the discussion was assured and permission to audio-record the discussion was requested. The focus groups were audio-recorded and transcribed verbatim.

### Data analysis

The transcripts from all interviews and focus group conversations have been coded in QSR Nvivo 10 [14]. Transcripts were read to gain an overall perspective. The data was analysed using inductive content analysis with constant comparison [15]. The objective of using a constant comparative method is to identify patterns in the data and discover relationships between ideas or concepts.

In a first step all transcripts were coded to reduce the text to meaningful and manageable parts (open coding). These parts were then summarized into more abstract categories (axial coding). Six out of 42 transcripts have been coded in parallel by two researchers. One of them carried out the interviews. Node trees have been compared and converged to a large extent. Differences between the two node trees have been discussed until one common node tree was agreed upon.

To maintain participant confidentiality, names of individuals, or institutions were deleted from the transcripts. Quotes were chosen to best illustrate the themes emerging from the data.

### Ethical approval

Ethical approval was obtained from the ethical committees of all participating hospitals and the central ethical committee of the University Hospital Leuven (No. B322201317189).

#### Findings

In this study, we identified the specific problems and unmet care needs for patients with severe burn injuries throughout the care process, both from the patients’ and health care professionals’ perspective. The results section of this paper is built around the four main care transitions burn patients encounter during the care process: referral and admission to the burn centre, discharge, return home and reintegration in social life (see Fig. 1). Note that discharge refers to discharge to another hospital ward or rehabilitation centre to avoid confusion with return home.

### Participants

Data from 57 respondents has been analysed: 29 patient interviews (see Table 2) representing 26 cases (i.e. for three cases adolescents as well as the parents were interviewed); 7 physician interviews, 4 interviews with representatives of patient organisations and 2 focus group conversations including data from 17 allied health professionals.

### Referral and admission to the burn centre

In order to optimize the care in the post-acute phase and the patient outcomes, an efficient and effective referral and admission to a burn centre is essential. Healthcare professionals stressed the importance of specialized dedicated multidisciplinary input and appropriate facilities during the acute phase to prevent problems (e.g. hypertrophic scars) in the post-acute phase or aftercare. In addition to the risk of worse outcomes, patients with severe burn injuries not treated in a burn centre during the acute phase have limited access to aftercare services and information.

Some patients experienced an immediate and professionally organized referral to a burn centre while others reported wrong assessments and physicians in general hospitals refusing to refer them to a burn centre, believing that they could handle them themselves. Patients report experiences of unnecessary intense suffering. Especially children and their parents sometimes had the impression they were not taken seriously.

### Discharge from the burn centre

#### Discharge protocol and procedures vary widely between burn centres

The discharge from the burn centre is considered as a crucial moment in the care process. Yet, most burn centres do not have a written discharge protocol. During interviews it was stressed that the average burn patient does not exist. Nevertheless, a harmonized protocol including minimal discharge criteria, does not contradict the requisite of tailoring discharge planning to patients’ needs. A discharge plan should be the result of a structured communication and information exchange involving the multidisciplinary team of the burn centre, the patient and his/her relatives and the healthcare professionals to which patients are referred.

Substantial variation in discharge procedures is observed. We identified three scenario’s regarding the organization of home care at the moment of discharge. In a first scenario, staff from the burn centre organizes the care at home by contacting primary care providers such as nurses and/or physiotherapists and scheduling ambulatory visits at the burn centre. Alternatively, staff from the burn centre provides names of primary care providers and the patient or a relative is expected to contact one of them to organize home care themselves. In a third scenario, patients do not receive any information regarding home care. Quite often patients reported that they had to organize home care themselves, often with the help of their personal network of family and friends.

The type of scenario actually occurring is closely related with the specific role definition of the ‘care coordinator’. In some centres, the care coordinators have a very restricted role (e.g. post-discharge wound care planning) while in other centres the care coordinator is responsible for the entire case-management of the post-discharge care plan.

#### Initiatives to foster good practices in discharge planning are not widely implemented

The data highlight some examples of good practices in discharge planning. However, these initiatives are local and mostly based on the good will of specific professionals, without being widely implemented nor structurally supported. In order to be sustained, they should be incorporated at the system level (i.e. national level).

An example is the use of a gradual discharge process (e.g. patients returning home for only one day, or during a weekend) to explore how those patients manage at home. A gradual discharge aims at preventing unanticipated problems at home. Yet, from the patients’ accounts we learned that the potential benefit of such temporary discharges is sometimes lost due to superficial evaluations upon return to the burn centre.

*‘When I came home the first time my apartment was a mess; nothing had changed since I left. Burn wastes, not to mention the curtains, half of the walls were filthy, it was dirty, it was incredible! So, I went back for the week-end but was unable to do the cleaning; therefore I left my apartment as it was. Fortunately, there is a big convenience store across the street. I bought ready-made meals so I only had to heat-it to have something to eat. When I returned to the hospital Sunday evening they asked me ‘Did it go well?’ then I said ‘It went pretty well, … yes,… but, … I lived all the week-end in a pigsty, cooking was nearly impossible because I could not properly use my fingers, etc. Next week-end, same story, and on Tuesday or Wednesday they let me go home.’*

#### Discharge towards step down units or rehabilitation units

Discharge from the burn centre does not necessarily mean that the patient returns to his previous place of residence. He may also be discharged towards another hospital ward (step down unit) or a rehabilitation centre.

Discharge towards a general hospital ward with medium or low care beds was perceived by the health care professionals as a useful intermediate step in the development of self-management skills, autonomy and self-care.

*‘We try to transfer patients from the burn centre to a general hospital ward to learn to function more autonomously, and go home after that.’*

Patients, however, described discharge towards step down units as extremely difficult evoking strong and ambiguous emotions, especially if the transition was not well prepared. Patients experienced the care at the step-down units of lower quality with an insufficient nurse-to-patient ratio.

*‘After discharge I was in a wing of the hospital with two nurses for forty rooms. Even when they would have extra arms and legs, and despite a lot of good intentions, these persons could not provide the same quality of service as in a specialized unit with twenty nurses for 5 patients’*

Patients also experienced discontinuity of care. Nurses working at general wards or rehabilitation units are not specifically trained in burn injuries, which sometimes leads to mistakes or contradictory information.

Discontinuity of care was a source of insecurity and distrust towards the nursing staff. Also, patients were struck by the low intensity of care, associated with the smaller relative number of staff. They missed the personal contact and strong ties they had acquired with the staff at the burn centre.

*’(it is like) …falling from six stars to only one, I mean…I was demoralized. […] I was probably the first severe burn patient because the staff seemed to be completely lost.’*

*“… it was a rehabilitation ward, thus, they did not have special training for severe burn patients… They made some mistakes, as we say. […] they used a wound dressing… with a piece of plaster on fresh skin. Incredibly big mistake! When the plaster was removed, the skin peeled off with the plaster. They made the same mistake two or three times.’*

### Return home

#### Ambulatory care in the hospital after discharge

In general a multidisciplinary team at the burn centre follows patients after discharge. This is highly valued by patients.

Some patients saw no other possibility than to return to the burn centre for ambulatory care, because of the burden of care such as bathing or care needing narcosis,, the lack of skilled home care professionals, the desire of patients to receive continuous high-quality.

The interviews with the healthcare professionals showed that there is large variability in the organization of aftercare services in terms of *duration of follow-up*, *intensity* (e.g. frequency of outpatient visits), and *disciplines involved* (e.g. in some centres patients only see the care coordinator and the plastic surgeon while in other centres there is a multidisciplinary consultation involving plastic surgeon, physiotherapists, orthosist, wound care nurse, psychologist). From the patient interviews some additional problems were identified.

Patients praise burn centres for their personal contact and human approach during the acute hospital stay. However, after discharge some patients and parents return to the centre for ambulatory care where they expect to see the care providers they know and trust, while in practice they really experience a discontinuity of care. In burn centres where care coordinators operate as case managers of the entire post-discharge care plan, some continuity of care is guaranteed. This is not the case for burn centres where care coordinators have another role (e.g. wound care nurse). In addition, patients and parents would especially value meeting the same physician during all ambulatory visits.

In addition to discontinuity of care, patients reported receiving contradictory information and impersonal care.

*’We have difficulties with the way the follow-up by physicians is organized. It’s always an assistant or junior doctor. You just have to be Lucky with the one in front of you. You cannot build-up a trusting relationship. I remember a doctor coming in the room and he said: “Tell me, what happened?” I thought: “Are you serious? After all this time you want us to tell our story?” Isn’t there something like a patient medical record? It does not give you the impression that this physician will be able to effectively evaluate whether the injuries evolve well.’*

Furthermore, some patients describe consultations as hectic, with staff constantly entering and leaving the room. Patients express the need to sit down for a moment, giving them the opportunity to ask questions and do the treatment afterwards.

*‘I found it [the consultation] a little bit hectic. During a consultation I would personally prefer to sit down at a table to talk and receive information. I always have a list of questions. I would like to have them systematically addressing all of the injuries.”*

#### Ambulatory care at home after discharge

Given the high-frequency and intensity of the required aftercare some patients prefer to receive care at home, by primary care providers (home nurses, physiotherapists).

Still in general, patients and healthcare professionals reported that the knowledge regarding burn-related care issues is limited among nurses, physiotherapists and psychologists outside the burn centres. There is a relatively small numbers of patients. Therefore allied health professionals get little benefits in return for an investment in extra trainings, unless they decide to specialize in the care for burn patients and orient their practice towards this group.

*‘The problem it is that we are turning in circles because caring for specific patients implies training. But training is expensive and when you are trained, patients are rare and geographically distant, and the fees are poor. That is the reason why the field actors do not wish to invest in training’*

The lack of a formal list of competent primary care providers makes it difficult for patients to find a competent nurse or physiotherapist.

*‘It would not be a bad idea to ask patients about experiences with a competent nurse to pass on her/his name. If someone in the same region needs care for burn injuries, that person can then more easily find an experienced professional.’*

Nevertheless interviewees (patients and healthcare professionals) note that there is a lot of goodwill among allied care providers to learn. The primary care givers (e.g. physiotherapists) sometimes visit the burn centre to learn how to treat the injuries of a specific patient. This is commendable since primary care givers often spend more time on the very laborious and time-consuming care for severely burned patients than they are paid for under the current fee-for-service system.

*‘But the problem is just that there is no specific billing code for burn injuries. We do not need one and a half or two hours for each patient, of course not. But there should be a billing code for each type of treatment. Now treatments are being done, but solely because of the good will of care professionals, I would say.’*

#### The crucial role of informal support after discharge

The burden for family members is heavy: friends and relatives take care of daily-life support, care, administrative tasks and transportation (e.g. to ambulatory consultations). As this is often very intensive, they sometimes need to scale back their professional activities through social leave or part-time work arrangements. The professional psychological support was evaluated positively: by the spouse to take care of the care burden, by the patient to accept being dependent. This helped to keep couples together despite the dramatically changed partnership relation.

*‘My wife received a medical certificate. She went to the GP and he gave her a certificate. She told her employer: ‘It is to help my husband’ because I could not cut my meat, I could not do anything, even washing myself was painful. My wife helped me during one month…’*

*‘Fortunately, we had a psychologist at the hospital, otherwise, I would dare to say we wouldn’t be a couple anymore.‘*

Patients acknowledge the initial reluctance to seek or accept psychological support. Some feared stigmatizing reactions, others did not acknowledge their need for psychological help until far in the aftercare process when they realized that their condition would not improve anymore and they should learn to cope with the remaining functional limitations and scars for the rest of their lives.

*‘At the start I did not really need it [psychological support]. It came afterwards. [laughs] Once I knew the consequences of the accident. Interviewer: What do you mean with afterwards? After you returned home? Interviewee: [sighs]. It is not so long ago. Since I know about the potentially permanent character of my functional limitations’*

#### Communication and information towards the patient

The interviews with patients and especially parents of severely burned children are infused with an insatiable and continuous need for information at each stage in the care process. They feel they were not informed enough: not at the right time, not in the right way.

On the one hand, patients appreciate that caregivers adapted the content of information to the moment in the care processproviding pieces of information at the right time or step in the care process, in order to avoid an information overload. This is often a deliberate strategy of care providers, since they experience that patients only receive and retain the information which is relevant and of immediate use in the current situation. On the other hand patients expect information regarding the future in general and the total care process in particular.

Although care providers often cannot predict the evolution of the injuries, nor the general condition of a patient, some patients do want to hear about all possible outcomes, problems and solutions. They want to know what to expect to reduce uncertainties and to give them the opportunity to prepare for certain problems, treatments or solutions and to be able to plan ahead. Examples from the interviews are: change in type of lotions, the need for pressure garnments, minimal duration of the leave parents need to care for their burned child.

Patients sometimes needed to be proactive to get information, but at the same time they were aware that they themselves had not the expertise necessary to ask the right questions. They also criticised that information was given during treatments rather than at a quiet moment sitting together around a table.

### What makes reintegration in social life difficult?

Patients with severe burn injuries are isolated from social life for months, sometimes even years. They are pulled away from their usual activities, their home, their family and friends. After hospitalization, they need to gradually pick up their former life, but with new bodily conditions. Scars, functional limitations, and fear of stigmatizing reactions often refrain them from functioning as before.

The chronic character of burn injuries is seriously underestimated by patients themselves and by the general public. At their return home patients experience difficulties in coping with their changed body and others’ reactions to this changed body. Severely burned patients often become aware of the chronic character of more limited body functions and discomfort only late in the rehabilitation process. At that point they need to start adapting their personal expectations and plans for the future.

*‘It is perhaps a silly detail, but at the start it is very difficult to estimate. You get a certificate for a three to six months leave and you think: “I will have a hard time during six months, but then it will all be over.” Over… now I know that with burn injuries it will never be over.’*

At work or at school, it is expected that since the person comes back, he is cured and is able to return to normal functioning. For successful reintegration, the process of adapting expectations of the patient and the people around him/her seems essential.

In addition to false expectations, daily care (e.g. putting on pressure garments, smearing with crèmes several times each day, etc.) and care appointments are very time consuming, hence restrain patients’ participation in social activities. This is especially true for children and adolescents. Lack of time may contribute to isolation and feelings of loneliness. In contrast, boredom was mentioned especially by older patients as an adverse consequence of severe burns. The range of activities is limited due to limited mobility.

To prepare both the patient and the “receiving environment” for re-entry, and to manage expectations occupational therapists visit patients’ homes, workplace or classroom. However, generally there is a lack of structured professional support for re-entry. Mostly the return to school or work is organized by the patients themselves and their relatives. The return to school was for some young patients facilitated by their parents together with dedicated teachers and the support of the burn centres. Examples are parents or teachers giving private lessons, teachers providing children with exercises and or a diary circulating among the other pupils, children who return to school for a reduced number of days a week or part-time with only mornings or afternoons. Especially children in primary school seem to be well supported and teachers seem to be very involved. In secondary school there seems to be less initiatives to involve the other pupils or to facilitate reintegration in school. Moreover, the provided facilities were not standard practice and depended on good intentions and private initiatives of individuals.

The accounts of the return to work vary from positive to shocking. Some patients got modified work or support from the trade unions, others were being dismissed and still others were incapable of returning to work. Some patients worry about future financial security for their family because of the drop of their own and/or their partner’s income. Conflicts with the insurance medical experts are also reported as a practical problem.

Finally, burn patients, especially those with visible disfiguration, often feel stigmatized by the reactions from others. They feel stared at and uncomfortable in public, which complicates reintegration. Some tend to retreat from public life and get isolated. Patients experience difficulties in coping with their changed body because of problems with the self-image and identity and with the (perceived) reactions of others. Patients are tired of telling people what happened, hide their scars and pressure garments with clothing or make-up. Some patients report stigmatizing behaviour or remarks from others, like bullying at school.

## Conclusion and discussion

Unmet care needs for patients with severe burn injuries were identified throughout the aftercare process. Both the perspectives of patients and health care professionals are integrated in the analysis. Individual face-to-face interviews and focus groups were used to explore patients’ views on and experiences with the care process and to explore the views of professional care providers and representatives of patient organizations on the care process.

This study generated several key findings.

There is a need for an integrated aftercare process for burn patients. Other countries organise care for burn patients through multidisciplinary networks. An example is the British Burn Care Networks where hospital-oriented networks are geographically organized and NHS Burn care service providers are structured in three levels of inpatient care that work collaboratively (level 1: *Burn centres* - geographically discrete wards offering the highest level of critical care; level 2: *Burn units -* separately staffed discrete wards targeting patients with a moderate level of injury complexity; level 3: *Burn care facilities -* general plastic surgical wards for the non-complex burn injuries). Burn care services work in collaboration with their local hospital emergency departments to ensure patients are accurately assessed and referred to the right specialized care provider [16]. The results from our study stress the importance to extent such networks beyond the hospital and also include primary care givers within the network structures. All Belgian structures involved in the care for burn patients could be integrated into a two-layered burn care network. A first level could be a Regional Burn Care Network built around the burn centre as a central node. A network should bring together all the care that is required, at the right time, in function of the current patient needs. It could include step-down units (for referral and back-referral), rehabilitation centres and a network of multidisciplinary primary care professionals. In addition, the network should also include general hospitals with emergency rooms to ensure adequate referrals and back referrals. A second level should be a National Network for Burns. The variability in care as well as the failure to implement locally developed good-practices on a wider scale emphasize the need for an overarching structure to monitor the regional networks. It should develop a number of transversal activities such as the development of referral standards, common guidelines, professional standards, uniform description and quality criteria for the care coordinator role, bundled payment agreements.

Second, not all patients with severe burn injuries are timely (or not at all) referred to burn centres. Therefore, all hospitals admitting emergency patients should use a straightforward uniform triage tool. This triage should allow the immediate referral of patients with severe burn injuries to a burn centre. In such a referral guide special attention should go to children (especially the very young ones) since they are a large and vulnerable group for which special competencies are required. The recently developed British NHS ‘National Burn Care referral Guidance’ can be a source of inspiration in this respect [16]. It proposes a triage procedure based on TBSA, depth site, mechanism (aetiology) of the burn and some other factors that may impact the severity and complexity of the burn. In the guidance there are thresholds listed as ‘refer’ (i.e. it is recommended that the patient is referred to the particular level of specialized burn services) or ‘discuss’ (i.e. in such cases a discussion should take place with a consultant of the burn centre/unit about a potential referral). The use of telemedicine (e.g. video-conferencing/sharing photographs) could be explored in an effort to facilitate these consultations [17, 18]. The same principle could be applied for the less severely burned patients (e.g. general practitioners (GP’s) consulting a plastic surgeon in a general hospital). In any case, providing a uniform triage tools is only an essential first step. A specific course could be developed to train physicians regarding the recognition, assessment, stabilization and transfer of the severely burned patient. The international applied EMSB-course (Emergency Management of Severe Burns course) could serve as an example [19, 20].

Third, there is a need for a uniform discharge procedure. The burn patient population is heterogeneous and therefore it will be important to design a harmonized protocol including minimal discharge criteria that can be tailored to the individual patients’ needs. This could be implemented through individual patient-centred care plans, accessible to all caregivers involved in the care for this patient. The discharge plan should be the result of a structured communication and information exchange involving the multidisciplinary team of the burn centre, the patient and his/her relatives and the healthcare professionals to which patients are referred [21]. Our study results show that such harmonized discharge protocols already exist in one or two centres in Belgium but that these good practices have not been disseminated to the other centres.

Fourth, burn centres filled in the function of care coordinator in divergent ways. In some burn centres, this person already takes up the role of discharge and/or case-manager with the global overview, initiating and coordinating the care plan. However, the variability in their role and responsibilities necessitates the development of a clear and uniform role description with corresponding quality criteria (e.g. educational level, minimal/maximal caseload) and objective, verifiable measures for follow-up (e.g. availability of individualized care plan, report of contact with primary care-givers; and up-to-date contact list of skilled primary caregivers). Depending on the context of the healthcare system evaluation is needed to determine whether it is desirable to create a 'discharge coordinator role' or to assign the 'discharge coordinator tasks' to someone within the team. After all, it is of utmost importance that these tasks are performed, not who performs them. In Belgium, however the former strategy (i.e. a ‘discharge coordinator role’) seems most suitable since there are already dedicated (but variably implemented) resources to this role.

Fifth, the lack of primary care givers (in particular nurses and physiotherapists) was identified as one of the main problem areas in the organization of aftercare services for patients with severe burn injuries. It is recommended to organize burn care specific training courses at the post-graduate level. The curricula of these training programs should be based on professionals standards such as the British standards for physiotherapy in burn care [22]. The health professionals fulfilling these training criteria should be eligible for an official accreditation granting them access to the reimbursement of specific burn care procedures and services. In addition, burn centres should produce, publish and maintain an inventory of allied health professionals that are eligible for specific reimbursements for the care of patients with severe burn injuries and refer their patients to these healthcare professionals. This will ensure that patients receive care from competent healthcare professionals. In addition, it will ensure a sufficient case-load to enable healthcare professional to build up experience in burn care.

Finally, the general public is not well informed about severe burn injuries. The general public has no idea about the burden of taking care for patients with those injuries over time. The chronic character of burn injuries is seriously underestimated. Also patients themselves underestimate the chronic character of burn injuries, especially at the beginning of the care process, but even after they returned home. Awareness campaigns could start growing awareness and reduce stigma.

The key findings should be interpreted in the light of the study limitations.

Patients with severe burns are a very heterogeneous group. In addition to minimal inclusion criteria (6–24 months post-burn; admission on a burn centre that meets the Belgian legal criteria) we used language, the burn centre which took care of the patient, age, gender, number of surgical interventions, visibility of the burns, psychosocial and/or financial problems as sampling criteria to take this heterogeneity into account. Still, some profiles of patients were difficult to find, especially adolescents and adults between 31 and 40 years. However, this reflects the demographic characteristics of the population of severely burned patients.

Patients were recruited with the help of the care coordinators of the six Belgian burn centres. They agreed to send invitations to a list of eligible patients. Biases could have been introduced by the care coordinators if they unconsciously privileged certain patients over others. In addition, the fact that, in a second step, patients themselves had to contact the research team could be a second source of bias as it introduced a barrier which the most vulnerable patients might not be willing or capable to surpass.

This sampling strategy, by default, limits the sample to patients being treated in a burn centre. By consequence there is no information available about patients with severe burn injuries that were not admitted to a burn centre. Also most healthcare professionals worked in one of the six burn care centres. Interviewing primary care givers could have enriched the data.

Finally, within the available timeframe, a total number of 29 patient (or parent) interviews were achieved instead of the planned 36. Nevertheless, during data analysis, the researchers felt that saturation was attained, this is the point at which no new information was emerging from the data.

## Abbreviations

TBSA: Total body surface area
FTE: Full-time equivalent

## Footnotes

1 Each burn centre was visited (June-October 2012) by a delegation of the research team to explore the domain and meet key-players in the field.
